# Detecting Target Objects by Natural Language Instructions Using an RGB-D Camera

**DOI:** 10.3390/s16122117

**Published:** 2016-12-13

**Authors:** Jiatong Bao, Yunyi Jia, Yu Cheng, Hongru Tang, Ning Xi

**Affiliations:** 1 Department of Hydraulic, Energy and Power Engineering, Yangzhou University, Yangzhou 225127, China; hrtang@yzu.edu.cn; 2 Department of Automotive Engineering, Clemson University, Greenville, SC 29607, USA; yunyij@clemson.edu; 3 Department of Electrical and Computer Engineering, Michigan State University, East Lansing, MI 48824, USA; chengyu9@msu.edu (Y.C.); xin@msu.edu (N.X.)

**Keywords:** object grounding, target object detection, object recognition, natural language processing, natural language control, robotic manipulation system

## Abstract

Controlling robots by natural language (NL) is increasingly attracting attention for its versatility, convenience and no need of extensive training for users. Grounding is a crucial challenge of this problem to enable robots to understand NL instructions from humans. This paper mainly explores the object grounding problem and concretely studies how to detect target objects by the NL instructions using an RGB-D camera in robotic manipulation applications. In particular, a simple yet robust vision algorithm is applied to segment objects of interest. With the metric information of all segmented objects, the object attributes and relations between objects are further extracted. The NL instructions that incorporate multiple cues for object specifications are parsed into domain-specific annotations. The annotations from NL and extracted information from the RGB-D camera are matched in a computational state estimation framework to search all possible object grounding states. The final grounding is accomplished by selecting the states which have the maximum probabilities. An RGB-D scene dataset associated with different groups of NL instructions based on different cognition levels of the robot are collected. Quantitative evaluations on the dataset illustrate the advantages of the proposed method. The experiments of NL controlled object manipulation and NL-based task programming using a mobile manipulator show its effectiveness and practicability in robotic applications.

## 1. Introduction

As assistants to human beings, robots are moving into more service oriented roles in human life, both in living and working. As a result, robots will more often be used by people with minimal technical skills. Controlling robots by natural language (NL) has attracted much interest in recent years for its advantages of versatility, convenience and no need of extensive training for users in the human–robot interactions. However, NL describes tasks from the human’s perceptive, which is usually different from the knowledge and perception of the robot. Therefore, a grounding mechanism is required to connect the NL representations with some specific representations which could be understood and executed by the robot.

There are usually two types of grounding problems. One is action grounding and the other is object grounding. For the action grounding—to transfer the actions described in the NL to some defined robot actions—A set of mapping rules could be predefined or learned, since actions considered for robotic systems are usually limited. For the object grounding which aims at correlating NL references of objects to the physical objects being sensed and manipulated, it is more complicated and always depends on the unpredictable environmental setups. The versatility, complexity and ambiguity of NL also make the grounding problems more challenging.

This work mainly explores the object grounding problem and concretely studies how to detect target objects by NL instructions using an RGB-D camera in robotic manipulation tasks. In particular, a simple yet robust vision algorithm is applied to segment objects of interest using an RGB-D camera. With the metric information of all segmented objects, the relations between objects are further extracted. The segmented objects as well as their relations are regarded as the basic knowledge of the robot about the environment. Since humans are more likely to employ object attributes (e.g., name, color, shape, material, etc.) to describe objects, the state-of-the-art machine learning algorithms are further employed to identify which attributes an object of interest has. The NL instructions that incorporate multiple cues for object specification are parsed into domain-specific annotations and stored in linked lists. The annotations from NL and extracted information from the RGB-D camera are matched in a computational state estimation framework to search all possible object grounding states. The final grounding is accomplished by selecting the states which have the maximum probabilities.

The contribution of this paper is three-fold: (i) we formulate the problem of NL-based target object detection as the state estimation in the space of all possible object grounding states according to visual object segmentation results and extracted linguistic object cues; (ii) an RGB-D scene dataset as well as different groups of NL instructions based on different cognition levels of the robot are collected for evaluation of target object detection in robotic manipulation applications; and (iii) we show quantitative evaluation results on the dataset and experimentally validate the effectiveness and practicability of the proposed method on the applications of NL controlled object manipulation and NL-based task programming using our mobile manipulator system.

The rest of this paper is organized as follows. Section 2 introduces the related works. Section 3 describes the overall formulation of the NL-based target object detection problem. The technical details are illustrated in Section 4. The experimental results and discussions are provided in Section 5. Finally, Section 6 gives conclusions and looks towards future work.

## 2. Related Work

The work presented in this paper falls under a specific area of NL-based human–robot interaction where humans and robots are situated in a shared physical world. It is critical for the robot and its partner to quickly and reliably reach a mutual understanding before actions can move forward. Due to significantly mismatched capabilities of humans and robots, NL-based communication between them becomes very difficult [1,2].

Firstly, NL describes environments and tasks on a highly discrete and symbolic level, which is usually different from the continuous and numerical representations of knowledge and perception of the robot. Therefore, many studies have been conducted on the NL understanding problem and employ a formal representation to represent the linguistic instruction as the intermediate medium such that the robot is able to understand the instructor’s intent. The formal representation employed by current NL controlled robotic systems can be generally classified into two groups: Logic expression and action frame sequences.

Logic expression uses formal language to model the given NL instructions. Matuszek et al. [3] designed Robot Control Language (RCL), which is a subset of typed *λ*-calculus to represent navigational instructions. Dzifcak et al. [4] used *λ*-calculus to model the given linguistic commands. Kress-Gazit et al. [5] translated structured English input into linear temporal logic formula. The generated logic expressions are either mapped to primitive actions or transformed into discrete event controllers.

Action frame methods extract key information, such as verbs, landmarks, locations, objects, etc., from the NL instructions and put specific information into corresponding slots. Chen and Mooney [6] trained a parser to parse the NL commands into predicate-argument frames that correspond to primitive actions. Stenmark et al. [7] used similar action frames. Misra et al. [8] decomposed the NL sentences into verb clauses. A verb clause is a tuple containing the verb, the object on which it acts and the object relationship matrix. Forbes et al. [9] implemented a set of robust parametrized motion procedures on a PR2 mobile manipulator and converted the NL sentences into intended procedures and their parameter instantiations. Rybski et al. [10] designed a task representation called Directed Acyclic Graph (DAG), which includes a preconditioned list of the behavior, the behavior and the link to the next behavior. The outputted formal representations are action plans in fact, and can be implemented by the robot sequentially. We also follow the schema of action frame method and mainly focus on representing the object references with multiple cues which is the main work part for parameter instantiations of intended procedures. The NL instructions will be processed into domain-specific annotations and stored in linked lists that are suitable for computation.

Secondly, the robot usually does not have complete knowledge about the shared environment. It would not be able to connect human language to its own representations with limited environment knowledge. For example, owing to the uncertainties of sensor systems and information processing algorithms, the object references in NL instructions can not always be accurately identified by the robot, which will result in a failed interaction. Most existing methods try to improve the visual perceptual abilities of robots in order to bridge the gaps of perception.

Typical methods attempt to develop more robust vision algorithms to accurately detect and identify physical objects. Many studies have been conducted on low-level feature based representations [11,12,13] or high-level semantic representations [14,15,16] of the situated environment. For example, MOPED [12] has been demonstrated to be a robust framework to detect and recognize pre-learned objects from point-based features (e.g., SIFT) and their geometric relationships. Schwarz et al. [13] extracted object features using transfer learning from deep convolutional neural networks in order to recognize objects and estimate object poses. Sun et al. [15] investigated how to identify objects based on NL containing appearance and name attributes. They employed sparse coding techniques to learn attribute-dependent features and identified the objects based on the classified attribute labels. Zampogiannis et al. [16] modeled pairwise spatial relations between objects, given their point clouds in three dimensional space. They showed the representation is descriptive of the underlying high-level manipulation semantics. Many other works [17,18,19,20] focused on detection of previously unknown objects without relying on preexisting object models. The attribute based representation method can also be used to describe detected unknown objects.

Other methods of improvement have focused on refining object segmentation and description through human–robot collaborations or active interactions with objects, considering the fact that a robot will inevitably misunderstand some aspect of its visual input or encounter new objects that cannot be identified. For instance, Johnson-Roberson et al. [21] proposed to refine the model of a complex 3D scene through combining state-of-the-art computer vision and a natural dialog system. Sun et al. [22] proposed to interact with a user to identify new objects and find the exact meaning of novel names of known objects, based on a hierarchical semantic organization of objects and their names. Bao et al. [2] proposed to detect unknown objects and even discover previously undetected objects (e.g., objects occluded by or stacked on other objects) by incorporating feedback of robot states into the vision module in the evolving process of object interaction. Some works [23,24] show that, without any previous knowledge of the environment, the robot can utilize spatial and semantic information conveyed by the NL instructions to understand the environment. In this paper, a simple yet robust vision algorithm is applied to segment objects of interest using an RGB-D camera. The segmented objects could be updated based on the work [2] if object interactions happen. With the metric information of all segmented objects of interest, the algorithm of identifying object relations are proposed and models for recognizing object attributes are learned.

Thirdly, after translating a NL instruction into formal representations and sensing a situated environment with feasible vision algorithms, the language part should be connected with the sensing part in order to achieve a successful object grounding. Object grounding is always addressed in the NL understanding model using deterministic or probabilistic methods. For example, Tellex et al. [25] decomposed a NL command into a hierarchy of Spatial Description Clauses (SDCs) and inferred groundings in the world corresponding to each SDC. Howard et al. [26] applied the Distributed Correspondence Graph (DCG) model to exploit the grammatical structure of language. A probabilistic graphical model was formulated to expresses the correspondence between linguistic elements from the command and their corresponding groundings. Hemachandra et al. [24] employed the DCG model in a hierarchical fashion to infer the annotation and behavior distributions. Forbes et al. [9] presented the situated NL understanding model which computes the joint distribution that models the relationships between possible parametrized procedures, NL utterances and the world states. This model is then decomposed to the situated command model and the language model that generates referring expressions. Only a few object properties such as location, size and color are incorporated. Hu et al. [27] proposed to employ the Spatial Context Recurrent ConvNet (SCRC) model as a scoring function on candidate object boxes for localizing a target object based on a NL query of the object. Spatial configurations and global scene-level contextual information are integrated into the network. Since they worked on the 2D image, the object relations are characterized in the machining learning based scene-level contextual model. In this paper, we propose to formulate the problem of NL-based target object detection as the state estimation in the space of all possible object grounding states according to visual object segmentation results. The objects of interest are segmented in the 3D space using an RGB-D camera. Multiple cues including object name, common attributes (i.e., color, shape and material) and object relations (i.e., group-based relations and binary relations) are investigated in object specification and incorporated in the formulation.

## 3. Problem Formulation

The problem is how to enable a robotic system to detect target objects in the physical world based on a human’s NL instructions. A NL instruction could contain words of object attributes (i.e., name, color, shape, material) and relations between objects (e.g., A is to the left of B, the rightmost and biggest object, etc.). Currently, we consider that one NL instruction specifies only one target object. For example, if a human says “*pick up the cup that is to the right of the leftmost block*”, there are two object references “*the cup*” and “*the leftmost block*” and the target object is “*the cup*”. The words of object attributes are “*cup*” and “*block*”, while the words of object relations are “*to the right of*”. Instead of exhaustively detecting objects in the whole scene, we choose to segment out the objects of interest from the unknown scene by holding a commonly used assumption that objects are placed on a planar surface. In addition, an object of interest could be comprised of more than one actual object.

Suppose that there are *M* objects of interest $\{Obj_{m}\},m=1,\cdots ,M$ segmented out from the current scene using the method in Section 4.1. By applying the NL processing method in Section 4.4, a NL instruction is parsed to *K* object attribute labels with corresponding object references $\{\langle l_{k}^{att},R_{k}\rangle \},k=1,\cdots ,K$ and *J* object relation labels with corresponding object references and landmarks $\{\langle l_{j}^{rel},R_{j},L_{j}\rangle \},j=1,\cdots ,J$, where $R_{k},R_{j},L_{j}\in \left\{X_{n}\right\},n=1,\cdots ,N$. In total, the NL instruction contains *N* object references $\left\{X_{1},X_{2},\cdots ,X_{N}\right\}$ among which the target object reference is also determined by the NL processing module. It will have *M* possible groundings for each object reference $X_{n}$,

(1) $$g\left(X_{n}\right)\in \left\{\delta \left(X_{n},Obj_{m}\right)\right\},m=1,\cdots ,M$$

where $\delta (X_{n},Obj_{m})$ means the object reference $X_{n}$ is grounded to $Obj_{m}$. In all, there are $M^{N}$ possible grounding results for all object references. We then maintain the object grounding belief, $b(g\left(X_{1},\cdots ,X_{N}\right))$, which is a probability distribution over all possible object grounding results. The goal is to find the possible object grounding result with maximum probability

(2) $$\hat{g}\left(X_{1},\cdots ,X_{N}\right)=\arg\max_{g(X_{1},\cdots ,X_{N})}b\left(g\left(X_{1},\cdots ,X_{N}\right)\right)$$

For each possible $g(X_{1},\cdots ,X_{N})$ where $X_{n}$ is supposed to be grounded to a specific object of interest in the physical world, we calculate its probability by estimating the likelihood if the corresponding physical objects of interest have specific attributes and relation labels

(3) $$\begin{array}{c} b\left(g\left(X_{1},\cdots ,X_{N}\right)\right)=\prod_{k=1}^{K}p\left(l_{k}^{att}|R_{k}\right)\prod_{j=1}^{J}p\left(l_{j}^{rel}|R_{j},L_{j}\right) \end{array}$$

where $R_{k}$, $R_{j}$ and $L_{j}$ will be substituted by their corresponding physical objects of interest, $p(l_{k}^{att}|R_{k})$ could be calculated by using the classifiers introduced in Section 4.3, and $p(l_{j}^{rel}|R_{j},L_{j})$ would be calculated using the Algorithms 1 and 2 introduced in Section 4.2.

## 4. Method Description

### 4.1. Segmenting Objects of Interest on the Planar Surface

**Figure 1:**
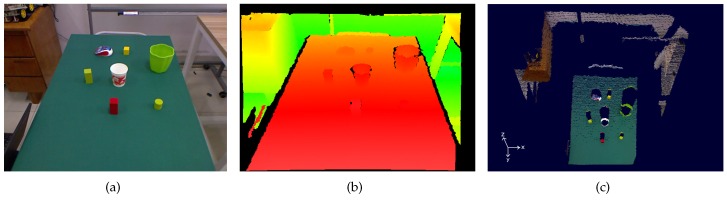
The RGB image (**a**), colored depth image (**b**), colored point cloud (**c**) of a sample scene.

A Microsoft Kinect RGB-D camera is employed to perceive the situated environment. As shown in Figure 1, this type of camera can capture 640 × 480 registered RGB images along with per-pixel depth information at 30 frames per second. As reported in [28], the random error of depth measurement increases with increasing distance to the sensor, and ranges from a few millimeters up to about 4 cm at the maximum range of the sensor. For each frame, a 3D point cloud can be generated from the RGB and depth data, providing the color information for each point.

We focus on segmenting 3D objects on a planar surface that could be the ground or a table. If the main plane could be detected, the 3D scene can be purged by removing points lying on and below the plane. The remaining 3D points could then be easily clustered spatially. Each cluster of 3D points represents a segmented object.

At the beginning, the point cloud of the scene is voxelized at a resolution of 3 mm to reduce the number of points in order to speed up related calculations. Thus, the volume of a voxel is 3 × 3 × 3 = 27 mm^3^. The parameter could be modified to achieve a balance between accuracy and speed according to the performance of the target computer. We then detect the dominating 3D support surface perpendicular to the gravity vector. Specifically, the normal vector of each sampled 3D point is calculated. Secondly, the gravity vector is estimated by finding the direction that is the most aligned to locally estimated surface normal directions at as many points as possible [29]. Thirdly, the points that have consistent normal vectors with the gravity vector are selected as the candidate support surface points. The dominating support surface is finally extracted, such that most of the selected points lie on the surface.

**Figure 2:**
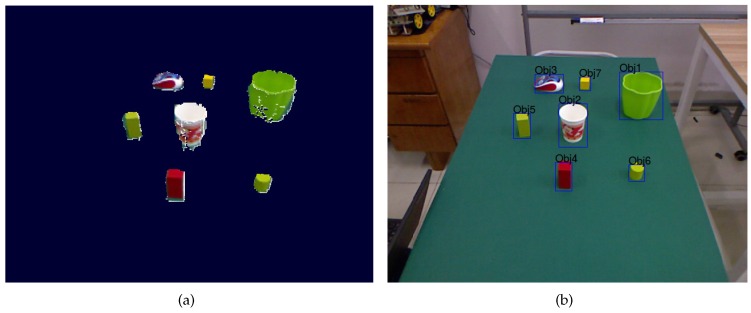
The object segmentation result of the sample scene. (**a**) shows the outputted RGB-D objects. For a better view, they are projected to the RGB image (**b**) where each object is associated with a bounding box and internally named.

After removing the points lying on and below the support surface, the remaining 3D points are clustered with a tolerance of 5 cm that specifies the minimal distance between any two objects. The clusters are considered as the segmented objects of interest. Since the objects are viewed from just a single point of view, we further estimate the hidden parts of the objects by exploiting the geometrical properties, and combine depth and color information for a better segmentation [30]. The object segmentation result of the sample scene is shown in Figure 2.

### 4.2. Identifying Relations between Objects

We categorize object relations into binary relations and group-based relations. In our current scenario, the binary relation labels include { *in front of, behind, to the left of, to the right of* } while the group-based relation labels include { *the leftmost, the rightmost, the foremost, the backmost, the highest, the lowest, the widest, the narrowest, the largest, the smallest* }. It can be seen that the binary relations capture the spatial relations between any two objects while the group-based relations capture the position, height, width and volume correlations among all objects. The raw position, height, width and volume values of the detected objects can be directly calculated from the corresponding point clouds. Especially, the volume of an object is computed by multiplying the voxel number with the voxel volume. The position of an object is represented by the centroid of its point cloud. All the metric values are finally scaled to meters.

To model the binary relations, the Algorithm 1, which outputs the likelihood of a binary relation type belonging to any two objects, is applied. The key idea is to make the spatial relation between two objects little vague for the human. For example, if the human says “*A is to the left of B*”, it means *A* is the nearest object that is to the left of *B* and it should not be in front of or behind *B* too much. The Algorithm 1 takes consideration of these implications. Specifically, every pair of objects (i.e., $O_{i},O_{j}$) are considered in the loop. The positions (x,y) of the two objects are compared and processed to the value which estimates the probability of a specific binary relation belonging to the objects. The probability values then constitute the corresponding relation matrix. The algorithm finally outputs four *M* × *M* relation matrices. In addition, *x* and *y* are defined in the coordinate framework of the camera as shown in Figure 1c.

The probability of an object that has a specific group-based relation type is estimated by using the Algorithm 2. Specifically, the volume *v*, height *h*, width *w*, position (x,y) of an object $O_{j}$ are compared with other M−1 objects by considering corresponding thresholds *δ*. Each type of semantic relation that $O_{j}$ has is counted and then averaged by the number M−1. This value estimates the probability of a specific group-based relation belonging to the object and constitutes the corresponding relation vector. The algorithm finally outputs ten *M* × 1 relation vectors.
**Algorithm 1** Algorithm of Calculating the Binary Relations.**Input:** the detected *M* objects *O***Output:** the *M* × *M* relation matrices *left*, *right*, *before*, and *behind*1: 2: **for** each object $O_{j}$ in *O*
**do**3: **for** each object $O_{i}$ in *O*
**do**4:  **if**
$O_{i}.x<O_{j}.x$
**then**5:   $left\left(i,j\right)\leftarrow exp(-10*\left(O_{j}.x-O_{i}.x\right))$;6:  7:  **else if**
$O_{i}.x>O_{j}.x$
**then**8:   $right\left(i,j\right)\leftarrow exp(-10*\left(O_{j}.x-O_{i}.x\right))$;9:  10:  **end if**11:  12:  **if**
$O_{i}.y<O_{j}.y$
**then**13:   $behind\left(i,j\right)\leftarrow exp(-10*\left(O_{j}.y-O_{i}.y\right))$;14:  15:  **else if**
$O_{i}.x>O_{j}.x$
**then**16:   $before\left(i,j\right)\leftarrow exp(-10*\left(O_{j}.y-O_{i}.y\right))$;17:  18:  **end if**19:  20:  probV←max(before(i,j),behind(i,j));21:  22:  probH←max(left(i,j),right(i,j));23:  24:  left(i,j)←sqrt(left(i,j)∗probV);25:  26:  right(i,j)←sqrt(right(i,j)∗probV);27:  28:  before(i,j)←sqrt(before(i,j)∗probH);29:  30:  behind(i,j)←sqrt(behind(i,j)∗probH);31: **end for**32: **end for**
**Algorithm 2** Algorithm of Calculating the Group-based Relations.**Input:** the detected *M* objects *O***Output:** the *M* × 1 relation vectors, *largest*, *smallest*, *highest*, *lowest*, *widest*, *narrowest*, *rightmost*, *leftmost*, *backmost*, and *foremost*1: 2: **for** each object $O_{j}$ in *O*
**do**3: **for** each object $O_{i}$ in *O*
**do**4:  **if**
$O_{j}.v>O_{i}.v$ and $O_{j}.v-O_{i}.v\geq \delta_{v}$
**then**5:   volume_larger_cnt←volume_larger_cnt+1;6: 7:  **else if**
$O_{j}.v<O_{i}.v$ and $O_{i}.v-O_{j}.v\geq \delta_{v}$
**then**8:   volume_less_cnt←volume_less_cnt+1;9: 10:  **end if**11:  **if**
$O_{j}.w>O_{i}.w$ and $O_{j}.w-O_{i}.w\geq \delta_{w}$
**then**12:   width_larger_cnt←width_larger_cnt+1;13: 14:  **else if**
$O_{j}.w<O_{i}.w$ and $O_{i}.w-O_{j}.w\geq \delta_{w}$
**then**15:   width_less_cnt←width_less_cnt+1;16:  **end if**17:  Count height_larger_cnt, height_less_cnt, horizon_larger_cnt, horizon_less_cnt, distance_larger_cnt, and distance_less_cnt in the similar way;18: **end for**19: largest(j)←volume_larger_cnt∗1.0/(M−1);20: 21: smallest(j)←volume_less_cnt∗1.0/(M−1);22: 23: Calculate highest(j), lowest(j), widest(j), narrowest(j), rightmost(j), leftmost(j), backmost(j), foremost(j) in the similar way;24: **end for**


### 4.3. Learning Object Attributes

We learn object attributes by constructing four kinds of classifiers (i.e., name, color, shape, material) in an off-line mode. For each kind k∈{Name,Color,Shape,Material}, the likelihood of the *i*-th label $l_{i}^{k}$ belonging to object *x* is calculated using a L2-regularized logistic regression model

(4) $$p\left(l_{i}^{k}|x\right)=\frac{\exp(g\left(W_{i}^{k}I_{x}\right))}{\sum_{j=1}^{N_{k}}\exp\left(g\left(W_{j}^{k}I_{x}\right)\right)}$$

where $I_{x}$ is the RGB-D feature vector of *x* learned by a state-of-the-art feature learning method [31] that uses hierarchical matching pursuit to generate a spatial max pooled sparse code for input images, $W_{i}^{k}$ is the learned model parameter vector for discriminating the *i*-th label from others, g(·) is the Sigmoid function, and $N_{k}$ is the number of attribute labels belonging to the attribute kind *k*.

Specifically, we first learn two general codebooks of size 1000 with sparsity level 4 on sampled 16 × 16 raw patches from RGB and depth object images, respectively. We name these two codebooks as color codebook and depth codebook. With the color and depth codebooks, the sparse codes of each pixel in the color and depth channels of an RGB-D object could then be calculated using batch orthogonal matching pursuit [32]. By employing spatial pyramid max pooling over the whole object image with 3 × 3, 2 × 2 and 1 × 1 partitions, the color and depth channels of the object can be finally represented by the two feature vectors, each of which has a size of 14,000 dimensions, respectively. Thus, an RGB-D object can be finally represented by a 28,000 dimensional feature vector. The parameters of the logistic regression models $\left\{W_{1}^{k},W_{2}^{k},\cdots ,W_{N_{k}}^{k}\right\}$ are finally optimized on the training samples using LibLinear [33].

### 4.4. Natural Language Processing

NL instructions express environment understanding from humans’ perspective and provide symbolic representations of object configurations. The main task of NL processing in this paper is to extract preferred object attributes and relations from the instructions, and to identify the corresponding variables denoting the object references. We start from NL processing and parse a NL instruction into the annotation which is the formal representation of object references.

After hearing the utterances, any state-of-the-art speech recognition modules could be applied to output the text type of instructions. In addition, we have integrated the CMU Sphinx [34] module in the system for speech recognition [35]. The NL instructions are then parsed using the Stanford parser [36]. The identified syntactic roles with corresponding words are processed into annotations using a priori rules that have been manually assigned or learned. An annotation is a set of object attributes and subspaces. We define object attribute as the category or appearance type (i.e., name, color, shape and material) with an attribute argument (e.g., cylinder (bottle), red (cuboid (block)), etc.), and subspace as the relation type with an object attribute argument (e.g., leftmost (cup), behind (blue (plate)), etc.).

**Figure 3:**
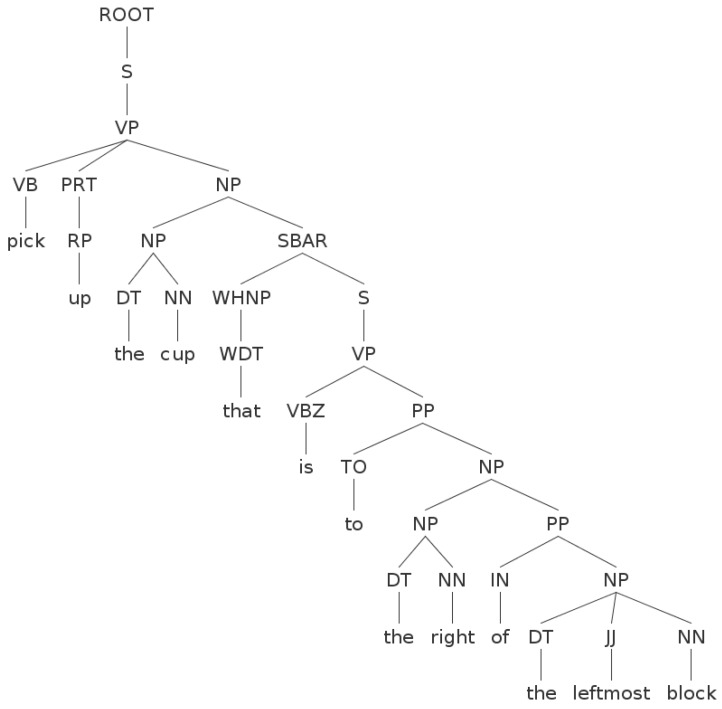
The parsed syntactic structure for the NL instruction “pick up the cup that is to the right of the leftmost block”. Please find the tags shown in the figure in the Penn Treebank [37] syntactic and part-of-speech tag-sets.

For the example scenario shown in Figure 2, a NL instruction could be “*pick up the cup that is to the right of the leftmost block*”. This instruction is parsed into the syntactic structure as shown in Figure 3. Then, the syntactic roles with corresponding words are processed into the annotation $\underset{4}{cup}\left(\underset{3}{right}\left(\underset{2}{leftmost}\left(\underset{1}{block}\right)\right)\right)$. Each part in the annotation is indexed ascendingly from inside to outside.

**Figure 4:**
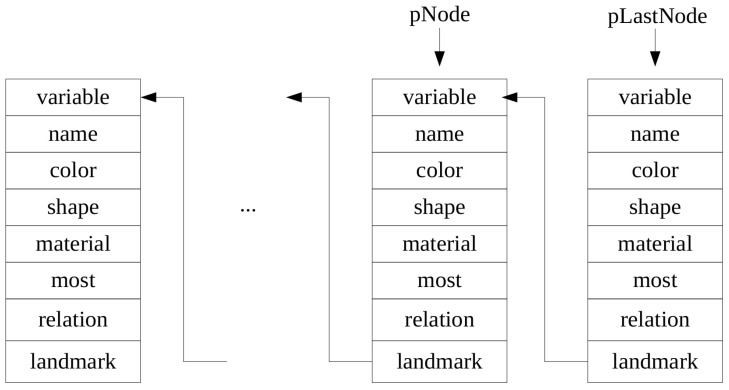
The linked list for storing information about object references.

Given an annotation, the Algorithm 3 is applied to associate variables to object references, assign attribute and relation labels to object variables, and identify if an object variable denotes a landmark or target object. This information is stored in a data structure (i.e., linked list), as shown in Figure 4. Specifically, the sorted parts in the given annotation are processed sequentially. If the current part is identified as an object name or a binary relation label, a new node of the linked list is created and associated with a new object variable. For the binary relation, the landmark variable in the new node should refer to the previous node. Other information is also updated accordingly. Otherwise, the annotation part is saved in the current node of the linked list. By searching in this data structure, the relationship between attribute or relation labels and object references that are used in Equation (3) could be quickly identified.
**Algorithm 3** Algorithm of Constructing Data Structure from Annotation.**Input:** the annotation *A* with parts sorted ascendingly by their indexes**Output:** the linked list with head pointer *pHead*1: 2: refObjCnt←0, pNode←NULL, pLastNode←NULL, pHead←NULL+;3: 4: **for** each sorted part *a* in *A*
**do**5: **if**
*a* is an object name **then**6:  **if**
pNodeisNULL
**then**7:   refObjCnt←refObjCnt+1;8: 9:   pNode←newLinkedListNode();10: 11:   pHead←pNode;12: 13:   $\left(*pNode\right).variable\leftarrow X_{refObjCnt}$;14: 15:  **end if**16: 17:  (∗pNode).name←a;18: 19:  pLastNode←pNode;20: 21: **else if**
*a* is a color label **then**22:  (∗pNode).color←a;23: 24: **else if**
*a* is a shape label **then**25:  (∗pNode).shape←a;26: 27: **else if**
*a* is a material label **then**28:  (∗pNode).material←a;29: 30: **else if**
*a* is a group-based relation label **then**31:  (∗pNode).most←a;32: 33: **else if**
*a* is a binary relation label **then**34:  refObjCnt←refObjCnt+1;35: 36:  pNode←newLinkedListNode();37: 38:  $\left(*pNode\right).variable\leftarrow X_{refObjCnt}$;39: 40:  (∗pNode).relation←a;41: 42:  (∗pNode).landmark←pLastNode;43: 44: **end if**45: **end for**


## 5. Experimental Results

### 5.1. Datasets of RGB-D Scenes and NL Instructions

To evaluate how well the proposed method detects target objects from various objects, we collected a dataset called RBT-SCENE which includes 100 scene images captured by a Microsoft Kinect RGB-D camera fixed on the top of the mobile base of our mobile manipulator [20]. RBT-SCENE has five parts, RBT-SCENE-2, RBT-SCENE-4, RBT-SCENE-6, RBT-SCENE-8 and RBT-SCENE-10. As show in Table 1, each part contains 20 scene images. Every scene image in these five parts respectively consists of 2, 4, 6, 8 and 10 daily objects which are randomly placed on the ground. In total, there are 600 physical objects.

For each scene image, we randomly marked one of the objects with a bounding box which indicates the target object people should refer to. We then collected NL instructions from 12 recruited people. In order to avoid issues with environmental noise affecting the reliability of speech recognition, we asked them to write down, for each scene image, three types of NL instructions that should be all sufficient to identify the target object, totaling 3600 NL instructions. It should be noted that we did not introduce much on the speech interface because this is neither the focus of this paper nor our contribution. As we know, much commercial software has achieved speech recognition with extremely high precision, thus they can be applied to realize the speech recognition function in real applications.

**Figure 5:**
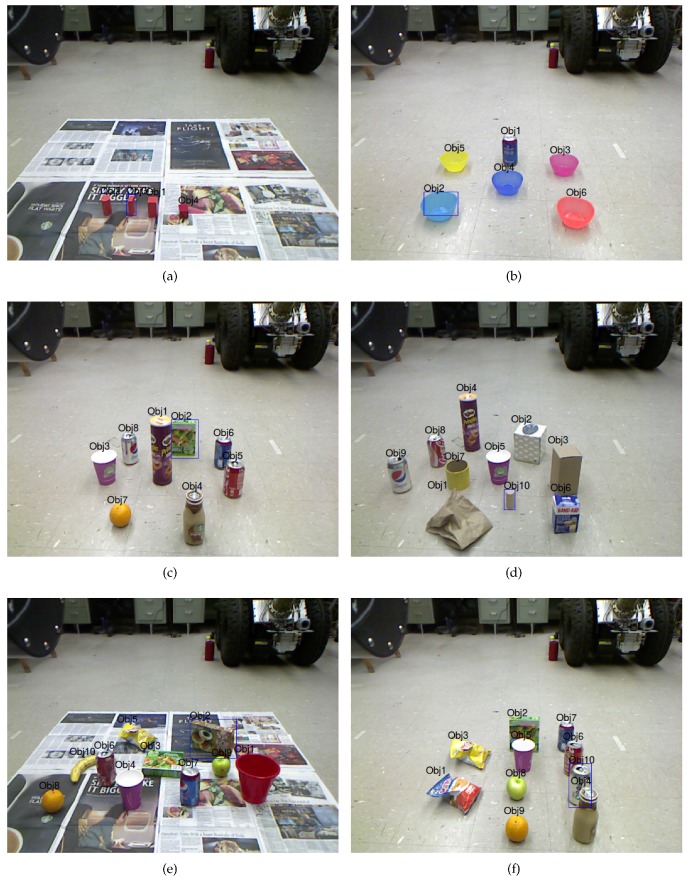
Example scenes along with three types of NL instructions. (**a**–**f**) show six different scenes. For each scene, the target object that people should refer to is marked with a bounding box. The examples of three types of NL instructions that employ object relations, object attributes (except name) and relations, and all cues are demonstrated respectively. (**a**) The object to the right of the leftmost object, the leftmost cuboid object or the leftmost cuboid block; (**b**) the leftmost and foremost object, the light blue object or the light blue bowl; (**c**) the backmost object, the green cuboid object or the green box; (**d**) the smallest object, the gray cylinder object or the gray block; (**e**) the backmost and rightmost object, the brown cuboid object or the backmost box; (**f**) the object behind the foremost and rightmost object, the silver cylinder object or the silver soda can.

The NL instructions, as well as the corresponding scene images, serve as the inputs for the robotic system to detect the target objects. The three types of NL instructions are based on the assumption that the robotic system is at three different levels of cognition, respectively. At the first level of cognition, we assume that the robotic system could understand some differences between objects but has no idea with object attributes. Thus, the first type of NL instructions should only contain object relation labels introduced in Section 4.2. The group of first type NL instructions is collected and named as NL-INST-1. At the second level of cognition, the robotic system could further understand some common attributes that objects have, i.e., color, shape and material, but has no knowledge about object names. Another underlying consideration is that robotic systems will inevitably come across new objects that have not been learned before. Therefore, the second group of NL instructions is collected and named as NL-INST-2 where object names are not allowed to be used and words of other attributes could be chosen freely. At the third level of cognition, the robotic system has further been taught some object names. All cues are allowed to be used in the third type of NL instructions, resulting in the third group of NL instructions named as NL-INST-3. The object names could also be chosen freely by the people. The three NL instruction datasets are listed in Table 2. Figure 5 shows some example scenes along with the NL instructions.

**Figure 6:**
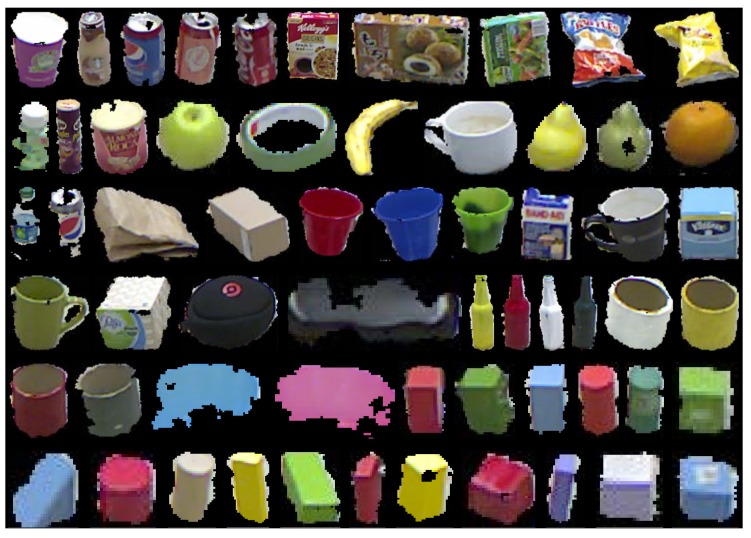
Example objects in our collected RGB-D object dataset.

We also collected the RGB and depth images of the daily objects that appear in the RBT-SCENE dataset, resulting in the RGB-D object dataset called RBT-OBJ. The collected samples of these objects are used for attribute learning. Figure 6 shows some example objects that have been segmented from the background. These datasets are available at [38].

### 5.2. Learning Object Attributes

We manually collected labels of object name, color, shape and material for every RGB-D object sample in the RBT-OBJ dataset. Since the number of physical objects collected by ourselves is limited, the attribute labels are not sufficient. Learning object names at large scale is not within the scope of this paper, so we only care about the objects that will appear in the target-domain scenarios (i.e., the RBT-SCENE dataset). In the RBT-OBJ dataset, 90% of samples per class are randomly selected as training samples for constructing the object name classifier while others are selected as the testing samples for evaluating its performance. The test set is randomly selected 10 times. The average accuracy of the object name classifier will be calculated over the 10 test sets.

Learning common attributes such as color, shape and material is very meaningful. Therefore, we supplemented the object color, shape and material labels using the Washington RGB-D object attribute dataset [22]. In the Washington RGB-D object attribute dataset, the RGB-D objects captured from the $30^{\circ }$ and $60^{\circ }$ elevation angles are used as the training set while the ones captured from the $45^{\circ }$ angle are deployed as the test set. All RGB-D object samples in the RBT-OBJ dataset are merged into the training set and the test set. Based on the training set and the test set, the color, shape and material classifiers could be constructed and evaluated.

The finally collected attribute labels to learn are shown in Table 3. It should be noted that learning object attributes, especially object names, is a never-ending task in many robotic applications. As it will be discussed in Section 5.3, even though we collect so many attribute labels in Table 3 for the scenarios in RBT-SCENE, the robotic system will still encounter new attribute labels for the same objects. This will definitely decrease the detection accuracies of the target objects.

**Figure 7:**
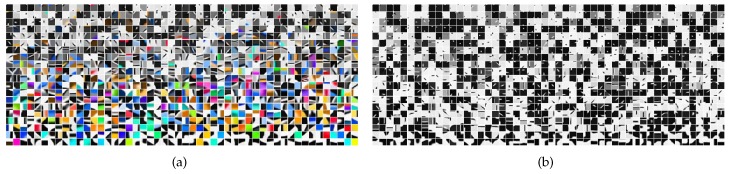
The learned color codebook (**a**) and depth codebook (**b**).

To implement the four classifiers, the color and depth codebooks were first learned as described in Section 4.3. Figure 7 shows the two codebooks. Each object can finally be represented by a 28,000 dimensional feature vector. The L2-regularized logistic regression models for the four kinds of classifiers were then learned individually. The recognition accuracies on the corresponding test sets for the classifiers are 94.12% (name), 94.69% (color), 92.53% (shape) and 94.47% (material), as shown in Table 4.

### 5.3. Target Object Detection Results

**Figure 8:**
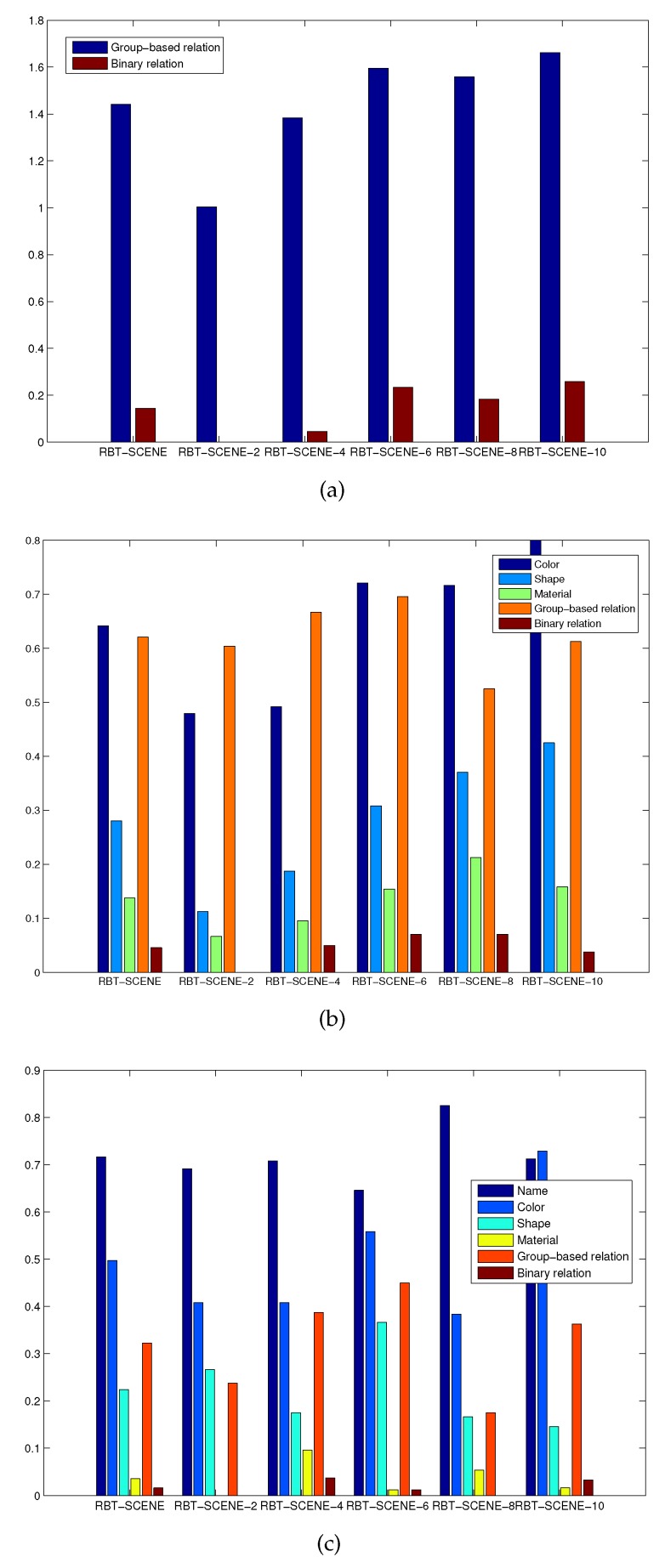
Occurrence frequency of different attributes and relation labels used by people for target object specification in NL-INST-1 (**a**), NL-INST-2 (**b**) and NL-INST-3 (**c**) corresponding to different subsets of the RBT-SCENE dataset.

**Observations.** We first evaluated which attributes and relation types people prefer to use for object specification under the constraint that the robotic system has different levels of cognition. For the NL instruction dataset NL-INST-1, Figure 8a shows the occurrence frequency of different relation labels in all the NL instructions for each subset of the RBT-SCENE dataset. The group-based relations are most frequently used while the binary relations are seldom used. The occurrence frequencies of the group-based and binary relation labels over the entire scene dataset are 1.44 and 0.14 times per instruction, respectively.

Figure 8b shows the occurrence frequency of different attribute and relation types for the NL-INST-2 dataset. It can be seen that as the scene images in the RBT-SCENE-2 and RBT-SCENE-4 subsets contain less than four objects, the group-based relation labels are used most frequently. It means that the group-based relation labels can locate the target object among a small number of objects with high probability. As the scene images contain more objects in the RBT-SCENE-6, RBT-SCENE-8 and RBT-SCENE-10 subsets, group-based relations becomes less discriminative while other attributes and relations should be combined. For the whole RBT-SCENE dataset, group-based relations and colors are the two most discriminative cues; materials are less used than shapes, and binary relations are rarely used.

Figure 8c shows the results for the NL-INST-3 dataset. Object name and color are two of the most frequently used cues. Group-based relation is still a useful cue that is more frequently employed than shape, material and binary relation. Besides, we found that people are accustomed to describe the object names at different semantic hierarchies. Some new words such as “coke can”, “pepsi can”, “food box”, “fruit”, etc., are encountered and they are different from the already learned labels listed in Table 3. A possible solution is to train classification models at different semantic hierarchies. Building semantic hierarchy trees to utilize already learned classifiers for recognizing objects with new names is also another possible way.

**Figure 9:**
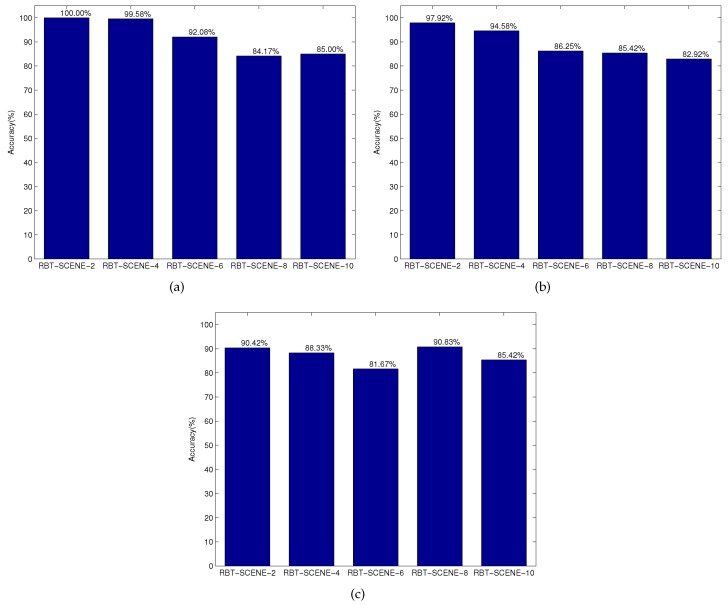
The accuracies of target object detection over different subsets of the RBT-SCENE dataset using three groups of NL instructions, NL-INST-1 (**a**), NL-INST-2 (**b**) and NL-INST-3 (**c**).

**Detection results.** We then evaluated how well the robot can detect the target objects given the three groups of NL instructions as well as the corresponding scene images. It should be noted that in our case one NL instruction specifies only one target object and the proposed method also outputs the one most possible object. Therefore, the number of false negatives (FN) equals to the number of false positives (FP), which means that the detection precision is the same as the detection recall. The detection accuracy reported below means precision, recall or F1-Measure. The detection results over each subset of the RBT-SCENE dataset using different NL instruction datasets are shown in Figure 9. The Matlab version source code of the target object identification module with processed NL and segmented objects are available at [38] for performance evaluation.

Figure 9a shows that when using the first group of NL instructions (i.e., NL-INST-1), the accuracy of target object detection decreases as the number of objects in the scene increases. This is not surprising, since humans will find it a little bit more difficult to accurately describe the target object in order to discriminate it from other objects. In other words, the NL sentences are more likely to become vague when the target object is located among a larger number of objects. In general, it can achieve a satisfactory identification accuracy of 92.17% which shows the effectiveness of object relations for locating objects.

Figure 9b shows the same trend of target object detection accuracy as more objects are involved in the scene when evaluating the second group of NL instructions NL-INST-2. It is true that when more cues are employed in the NL instructions, the robot should identify all referred attributes and relations, resulting in a relatively lower identification accuracy of 89.42%. Besides, we found that people may have ambiguous understanding of some attributes. For instance, the color “gray” and “brown” are always used with the same meaning. The ambiguities that exist in NL instructions would also decrease the identification accuracy.

Figure 9c shows that when evaluating the third group of NL instructions NL-INST-3 where people mainly deploy object name and color for object specification, there is little impact of object numbers on target object detection accuracy. As referred to above, many new object names appear in NL-INST-3, especially its subset corresponding to the scene dataset RBT-SCENE-6, thus the target object identification accuracy is relatively low. The average identification accuracy can still achieve 87.33% across all the scene datasets.

Table 5 reports the running time of some key modules of our current single-threaded C++ implementation of the proposed method for a typical 640 × 480 indoor scene RGB-D image. It runs on a 2.4-GHz dual-core 64-bit Linux laptop with 16 GB of memory, and is evaluated on the above NL instruction dataset as well as the RGB-D scene dataset. Basically, the overwhelming majority of computation is spent on the visual segmentation of objects of interest. The more complicated a scene is, the more 3D points should be processed. Since the structure of NL instruction is relatively simple, the parsing process takes negligible time. Besides, it takes about 0.91 s to identify the target object, where extraction of object features, and searching and estimating in object state space are a little bit time-consuming. Overall, it requires around 1.88 s to process an RGB-D frame using our current computing hardware. We believe that the codes could be optimized to achieve the real-time performance.

**Discussions.** Object relation is a very important cue for describing objects. This investigation is based on the consideration that, in the early learning stage of children, they cannot tell the object names and attributes, but can tell the differences between objects. However, only employing this cue for object specification is not the natural way for users, but is in fact the complicated way. The advantage is that when a robotic system has a low level capability of cognition, users can deploy this robust cue to command it to pay attention to the intended object and teach it new information about the object. When a robotic system has no ideas with new objects or new object names, employing common attributes of objects, as well as object relations, is a natural and good choice. This is because common attributes of objects have less diversities than the object names have and people are likely to use a relatively small set of color, shape and material labels. The small set of object attributes could be learned using the state-of-the-art machine learning algorithms. Employing object names and colors for object specification is the most natural and favorite way for humans. The key is whether the robotic system can deal with the diversity of object names and recognize the real objects with these names. Learning and recognizing new objects and object names in the robotic systems is a never-ending task.

Visually detecting target objects by NL is a systematic problem which involves three aspects: NL processing/understanding, visual sensing, and object grounding. As referred to before, a main challenge is the mismatched perception capabilities between humans and robots. Enhancing the visual sensing capabilities of robots will definitely improve the overall performance and extend application domains. The scenarios shown in the dataset are mainly collected for evaluation of the proposed object grounding method, since the object numbers, object types and various relations between objects could be quickly configured. We utilized the RGB-D camera for object detection and recognition. Based on this work and our previous work [2], we could currently deal with the separated objects supported by a planar surface, the stacked objects, and the occluded objects. It would be predictable that, in the event of performing novel tasks in unknown environments, the robots would still suffer the misunderstanding of complicated and unpredictable object setups. It is true that generic object detection and recognition is an open challenge in the research areas such as computer vision, robotics, etc. Studying visual sensing algorithms for general purpose is beyond the scope of this paper. We believe that any progress in computer vision algorithms and sensors will benefit the robotic applications in real scenarios.

In robotic applications, the common way is firstly to determine target-domain scenarios. Then, vision sensors could be chosen and visual algorithms be developed accordingly. Any state-of-the-art computer vision algorithms could also be applied in the system. Another possible solution is that the robotic system could report exceptions to human partners when new situations appear, such that human partners could teach it new knowledge about the environments according to its limited knowledge. In the following subsections, we will show two kinds of real applications in specific target domains based on the current capabilities of our robot.

### 5.4. Application on NL Controlled Object Manipulation

**Figure 10:**
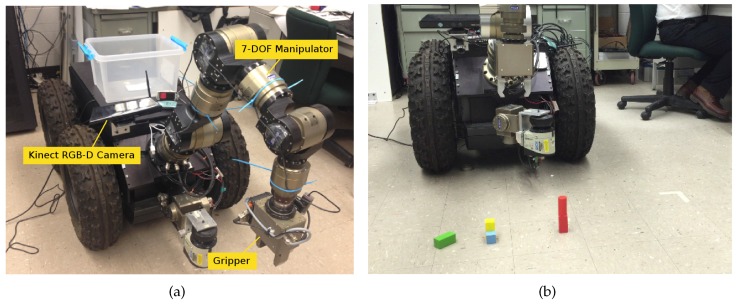
Our mobile manipulator (**a**) and an environmental setup (**b**).

We implemented and tested the proposed NL-based target object detection method in our mobile manipulator system [20,35] for object manipulation, as shown in Figure 10a. It consists of a 7-DOF Schunk LWA3 manipulator and a nonholonomic mobile base. A gripper is mounted on the end of the seventh link. A Microsoft Kinect RGB-D camera fixed on the top of robot base is used to perceive the environment, especially for the purpose of detecting target objects. The core library *Nestk* [30] was used for developing vision algorithms. For each detected object to be manipulated, the grasping position and gesture could be estimated from the corresponding point cloud. For reasons of simplicity, we choose to move the gripper down vertically to the top center of an object to attempt a grasp. Before manipulating the detected objects, the transformation from the coordinate frame of the camera system to the one of mobile manipulator is calibrated. The robot status is sensed by the on-board sensors. The robot information, including the end-effector position and gripper fingers’ position and force, are calculated based on the kinematic model, encoder readings and tactile sensor readings. All modules and algorithms are implemented on the on-board computer.

In the experiment, two basic actions *PickUp(src)* and *PutDown(dest)* were pre-programed. The robot was commanded to perform block manipulation operations in the scene shown in Figure 10b. The environmental setup is comprised of five blocks where two red blocks are stacked together and a blue block is occluded by a yellow block. The previous work [2] was employed to discover the stacked and occluded objects. The NL instructions are “*Pick up the red object. Put it down to the rightmost green block. Pick up the yellow block. Put it down to the ground that is in front of the blue block. Pick up the blue block. Put it down to the leftmost red block*”. This kind of experiment could also be regarded as a generic form of work piece assembly in specific target domains.

**Figure 11:**
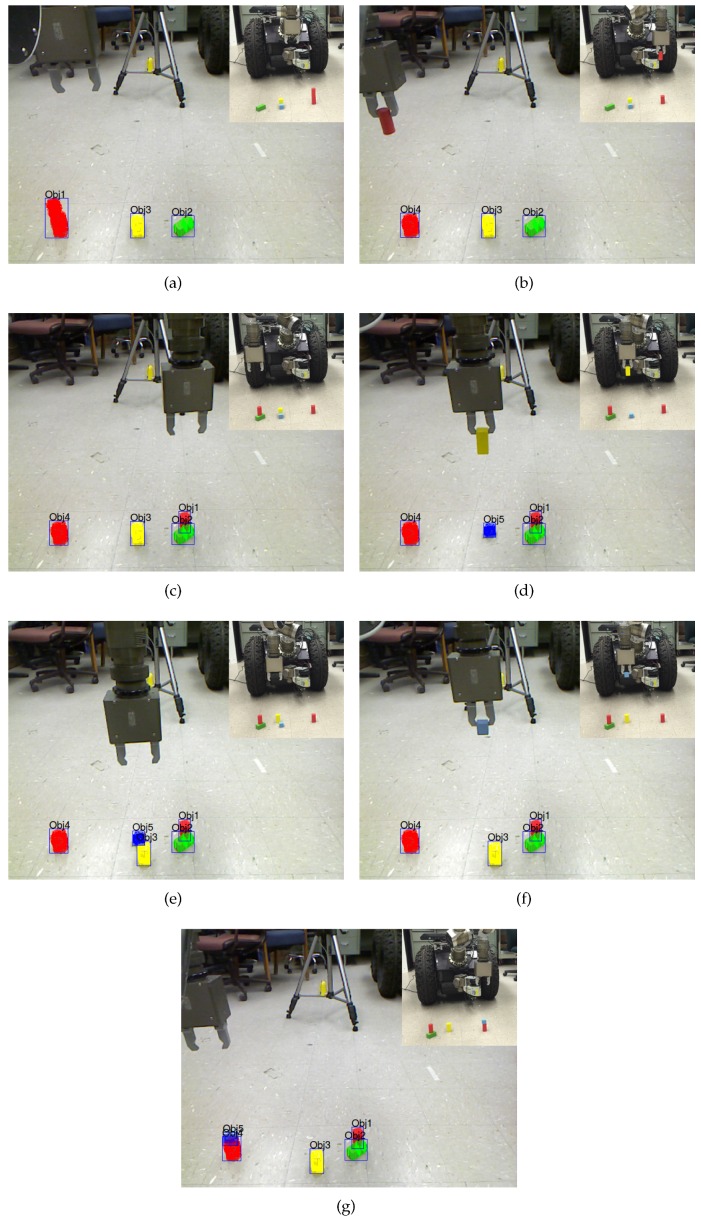
The block manipulation process at discrete time steps (**a**–**g**). At each time step *t*, the segmented objects are updated after executing the NL instruction at time step *t* − 1. (**a**) Time step 0: Pick up the red object; (**b**) Time step 1: Put it down to the rightmost green block; (**c**) Time step 2: Pick up the yellow block; (**d**) Time step 3: Put it down to the ground that is in front of the blue block; (**e**) Time step 4: Pick up the blue block; (**f**) Time step 5: Put it down to the leftmost red block; (**g**) Time step 6.

The execution progress of the task is shown in Figure 11. At the beginning, the objects on the ground were segmented and internally named by using the developed vision algorithm, as shown in Figure 11a. When the robot started to execute the first NL instruction, it translated the NL sentence into actions and annotations. The action words “*pick up*” were mapped into the predefined action *PickUp(src)*. The annotation *red(object)* was used for locating the target object. After grounding the red object to $Obj_{1}$, the action was instantiated to *PickUp($Obj_{1}$)* that can be directly executed by the robot. The representation of the scene was then updated accordingly [2], as shown in Figure 11b. The robot then processed the next NL instructions in the similar way. The rightmost green block, the yellow block, the blue block and the leftmost red block were all successfully grounded to $Obj_{2}$, $Obj_{3}$, $Obj_{5}$ and $Obj_{4}$, respectively. In addition, the action words “*put down*” were mapped into the predefined action PutDown(dest). The corresponding results are shown in Figure 11c–g. It can be seen from the experiment that, following instructions from humans, the robot could easily locate the target objects by using its own limited knowledge and complete the whole task. A video illustrating the block manipulation process is available at [38].

### 5.5. Application on NL-Based Task Programming

With the capability of detecting target objects by NL, the robot could also learn novel tasks from interactions with humans [39]. In this type of experiments, the subjects are firstly asked to teach the robot to accomplish the task by NL instructions under an environmental setup. The teaching process is performed in a human–robot collaborative teaching mode in which the human will not give the robot next NL instruction until the robot finishes the previous one. After the teaching is finished, robot learning is performed. Afterwards, we will give the robot the similar tasks under different environmental setups and ask the robot to execute the tasks according to the already learned knowledge.

**Figure 12:**
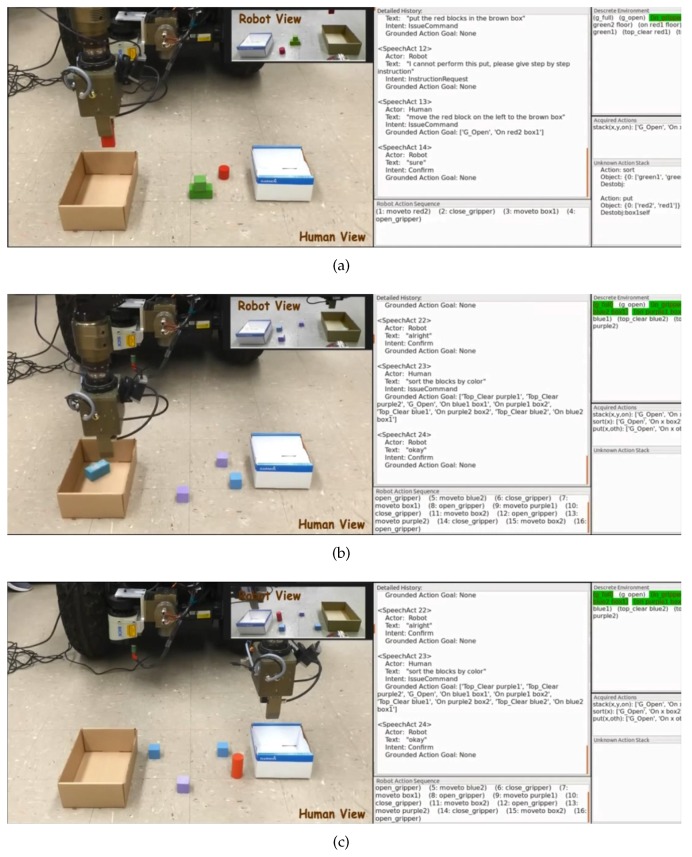
A demonstration of programming robots’ “sort” task by NL. (**a**) shows the scenario for teaching the robot a new task of sorting by color; (**b**) is a new environmental setup for testing; (**c**) shows that the robot could generalize the learned knowledge to the task of sorting by shape.

Figure 12 shows a demonstration of programming robots’ “sort” task by NL. At the very beginning, the human commands the robot to sort the blocks by color as shown in Figure 12a. The left part of the figure shows the actual scenario while the right part shows the graphical user interface of speech recognition and NL processing results. Since the robot has not learned the task before, it responds to the human with “*What do you mean by this sort?*”. Therefore, the human gives it step-by-step instructions. The following instructions “*Move the red block on the right to the brown box. Move the red block on the left to the brown box. Put the green blocks in the white box.*” are performed sequentially. In the process of instruction execution, object grounding should be performed as well based on the proposed method. After hearing the utterance “*Now you achieve the sort action*”, the robot starts the learning process and generates the system goal states for representing the new task. Then, the robot is given a similar task in the new environmental setup as shown in Figure 12b. According to their corresponding system goal states, the robot can correctly formulate the practical goal states for the “sort” task in new environmental setups. Furthermore, the action scheduling can successfully generate correct action sequences to achieve the whole task. The learned task knowledge can also be generalized to other similar tasks, such as “*Sort the blocks by shape*” as shown in Figure 12c. A video illustrating the task programming process is available at [40].

## 6. Conlusions

This paper mainly investigates the object grounding problem and specifically studies how to integrate the NL cues and visual sensing information into a computation framework. We formulate the problem of NL-based target object detection as the state estimation in the space of all possible object grounding states according to visual object segmentation results and extracted linguistic object cues. We have collected an RGB-D scene dataset, as well as different groups of NL instruction for target object detection, using different combinations of object cues, based on the considerations that a robotic system could have different levels of sensing capabilities. The evaluations on the dataset, as well as two applications on NL-based object manipulation and task programming, show the effectiveness and potential of the proposed method.

However, the structure of the NL instructions used in our experiments is simple, since the current applications care about the objects that are placed in a relatively small space. We would explore more flexible structures of NL instructions for specifying objects to be sensed and manipulated within a large scale of space, according to new application needs where complex target-domain scenarios may be involved. This will also require the robot to enhance its visual sensing and understanding capabilities. In addition, identifying target objects by the diversities of object names is also a challenging yet significant problem since humans are more likely to employ object names to specify the objects. Future work will also address these challenging problems.

## Figures and Tables

**Table 1:** RGB-D scene dataset characteristics.

RGB-D Scene Dataset	Scene Number	Object Number in Each Scene	Total Object Number
RBT-SCENE-2	20	2	40
RBT-SCENE-4	20	4	80
RBT-SCENE-6	20	6	120
RBT-SCENE-8	20	8	160
RBT-SCENE-10	20	10	200
RBT-SCENE	100	/	600

**Table 2:** NL instruction dataset characteristics.

NL Instruction Dataset	Corresponding Scene Dataset	Instruction Number	Object Cues in Instructions
NL-INST-1	RBT-SCENE (100 scenes)	1200 (100 scenes * 12 people)	Object relation labels
NL-INST-2	RBT-SCENE (100 scenes)	1200 (100 scenes * 12 people)	Object attribute (except name) and relation labels
NL-INST-3	RBT-SCENE (100 scenes)	1200 (100 scenes * 12 people)	Object attribute and relation labels

**Table 3:** The object attribute labels to learn.

Attribute Type	Labels
Name	Apple, Bag, Banana, Block, Bottle, Bowl, Box, Bucket, Can, Cup, Jar, Mug, Orange, Pear, Tape
Color	Red, Orange, Yellow, Green, Dark Green, Blue, Light Blue, Purple, Pink, Brown, Black, White, Gray, Silver, Transparent
Shape	Arch, Bag, Bowl, Circular, Cuboid, Cylinder, Ellipsoid, Rectangle, Triangular
Material	Ceramic, Fabric, Foam, Glass, Metal, Natural, Paper, Plastic, Wood

**Table 4:** Recognition accuracy of object attributes on the test set.

Name	Color	Shape	Material
94.12%	94.69%	92.53%	94.47%

**Table 5:** Running time of the proposed method.

Segmentation of Objects of Interest	NL Processing	Target Object Identification	Overall
0.94 (±0.43) s	0.03 (±0.01) s	0.91 (±0.30) s	1.88 s
